# A Tutorial on Implantable Hearing Amplification Options for Adults with Unilateral Microtia and Atresia

**DOI:** 10.1155/2014/703256

**Published:** 2014-06-02

**Authors:** Joannie Ka Yin Yu, Lena Lai Nar Wong, Willis Sung Shan Tsang, Michael Chi Fai Tong

**Affiliations:** ^1^Department of Otorhinolaryngology, Head & Neck Surgery, The Chinese University of Hong Kong, 6/F Clinical Science Building, Prince of Wales Hospital, Shatin, New Territories, Hong Kong; ^2^The Institute of Human Communicative Research, The Chinese University of Hong Kong, Hong Kong; ^3^Division of Speech & Hearing Science, Faculty of Education, The University of Hong Kong, Hong Kong

## Abstract

*Background.* Patients with unilateral atresia and microtia encounter problems in sound localization and speech understanding in noise. Although there are four implantable hearing devices available, there is little discussion and evidence on the application of these devices on patients with unilateral atresia and microtia problems. *Objective.* This paper will review the details of these four implantable hearing devices for the treatment of unilateral atresia. They are percuteaneous osseointegrated bone anchored hearing aid, Vibrant Soundbridge middle ear implant, Bonebridge bone conduction system, and Carina fully implantable hearing device. *Methods.* Four implantable hearing devices were reviewed and compared. The clinical decision process that led to the recommendation of a device was illustrated by using a case study. *Conclusions.* The selection of appropriate implantable hearing devices should be based on various factors, including radiological findings and patient preferences, possible surgical complications, whether the device is Food and Drug Administration- (FDA-)/CE-approved, and the finances. To ensure the accurate evaluation of candidacy and outcomes, the evaluation methods should be adapted to suite the type of hearing device.

## 1. Introduction

Difficulties in sound localization and speech understanding in noise have been underestimated in adults with unilateral atresia [1]. The amplification options for atretic canal patients are limited; this is because conventional amplification is not a treatment option due to the absence of an external ear canal. In addition, the cosmetic concerns of a conventional bone conduction hearing aid often lead to a relatively low compliance rate. Implantable hearing aids have been developed recently as alternative treatment options to fill in these gaps.

There are four commercially available implantable hearing devices available as rehabilitative options for patients with unilateral microtia and atresia. They are

- Percutaneous osseointegrated bone anchored hearing aid (p-BAHA), for example, Cochlear BAHA by Cochlear Bone Anchored Solutions AB and Ponto by Oticon Medical AB, Sweden;
- Vibrant Soundbridge (VSB) middle ear implant system (MED-EL, Innsbruck, Austria);
- BoneBridge bone (BB) conduction system (MED-EL, Innsbruck, Austria);
- Otologics MET Carina fully implantable hearing device (FIHA) (Otologics LLC, Boulder, CO, USA).

All of these devices need to be implanted surgically. These devices are expected to benefit patients with moderate to severe hearing, regardless of their type of hearing loss, that is, sensorineural, conductive, or mixed. However, these devices differ in terms of the sound transmission pathway, and some, such as the FIHA, are suitable for adults only.

### 1.1. The Percutaneous Bone Anchored Hearing System (p-BAHA)

The bone anchored hearing aid (BAHA) was first introduced in 1977 as an alternative hearing rehabilitation option for patients with conductive hearing loss. Later developments expanded the indications to include single-sided deafness and mixed hearing loss. The BAHA hearing system incorporated the concept of osseointegration discovered by Professor Brånemark, enabling improved comfort and fine sound quality. It represented a major milestone in the evolution of bone conduction hearing amplification [2]. Two commercially available systems of the BAHA hearing devices include the Cochlear BAHA by Cochlear Bone Anchored Solutions AB or the Ponto by Oticon Medical AB, Sweden. These devices are FDA-approved for children of the age of 5 and above; for younger children, a BAHA soft band will be applied.

The surgical procedure for the BAHA is relatively simple in comparison with other implantable devices. BAHA is placed in the mastoid bone. Local anesthesia could be used for the majority of the adult cases. The most obvious benefit for the BAHA system is its surgical simplicity; it bypasses the outer and middle ear pathology and stimulates the inner ear effectively via direct bone conduction, which is particularly well suited for patients with aural atresia. However, despite years of technological advancement, the problem of postoperative periabutment wound infection has remained at approximately 5%, which results in a significant negative impact on the clinical application.

### 1.2. The Vibrant Soundbridge Middle Ear Implant

An alternative to p-BAHA is the Vibrant Soundbridge middle ear implant system (VSB) (Med-El, Innsbruck, Austria). The Vibrant Soundbridge (VSB) is a direct-drive, partly implantable middle ear hearing device which was approved by the FDA in 2000. It is intended for patients of the age of 3 and above with a mild to severe degree of hearing loss, with their bone conduction thresholds at or not worse than 65 dBHL across frequencies.

The VSB system is comprised of two parts, namely, an internal component of the VSB called the Vibrating Ossicular Prosthesis (VORP) and an external audio processor (AP). The VORP consists of an internal coil, a magnet to hold the Amadé audio processor over the implant, a demodulator, the conductor link, and the floating mass transducer (FMT). The VORP is implanted by a surgical procedure in which the FMT is attached to a vibratory structure in the middle ear either via a round window approach (round window vibroplasty (RWV)) or via ossicular chain coupling [3]. Intraoperative electrocochleography is needed to locate the best orientation of the floating mass transducer. The details of the electrocochleography procedure are available in the guidelines written by Radeloff and colleagues [4]. The AP is about 1.2 inches in diameter and contains a microphone, which picks up sounds and converts them into electrical signals that can be transmitted across the skin to be received by the implanted internal receiver of the VSB.

Benefits from the VSB device include, firstly, the provision of a unilateral stimulation to the atretic ear, which is beneficial for those with unilateral hearing loss. Secondly, there is no pin tract problem as in BAHA. In addition, the patient's satisfaction with the sound quality and the improvement in speech intelligibility are higher and could be sustained even after five to eight years of implant use [5, 6]. However, because the VSB surgery involves the manipulation of the fragile ossicle bones, there are therefore possible risks of surgical trauma and irreparable sensorineural deafness. Other potential complications include postsurgical displacement of the implant due to development of scar tissues, taste disturbance, or damage to the chorda tympani nerve [5–7]. Lastly, magnetic resonance imaging (MRI) is contraindicated in patients with VSB device.

### 1.3. The Bonebridge Bone Conduction Implant

The Bonebridge (BB) (Med-EL; Innsbruck, Austria) is another feasible option for children at age of 5 years and above who have conductive, mixed hearing loss or single-sided deafness with bone conduction thresholds at 45 dBHL or better at 0.5, 1, 2, and 3 kHz. It is a semi-implantable bone conduction implant hearing device consisting of an external audio processor (AP) worn behind the ear and a bone conduction implant (BCI) positioned surgically under the skin. The acoustic signals are recorded by the microphones of the AP, which converts sounds into electrical signals, and these are then transferred to the BCI. The BCI then converts the electrical signals to mechanical vibrations on the mastoid bone and thus the inner ear is stimulated via bone conduction.

Several advantages have been noted with the BB. Firstly, the BB offers a bilateral stimulation that is similar to the BAHA. Thus, it is especially beneficial for those with bilateral hearing loss in that they will need to wear only one device to achieve desirable outcomes. Secondly, the BB does not require a good middle ear structure for the coupling of the device; thus it is suitable for patients with middle ear pathologies and who are not feasible for VSB implantation. Thirdly, the risks associated with the use of MRI are reduced, compared to the VSB. The BB allows MRI to be done up to 1.5 Tesla.

Although the BB seems to be a better device in terms of surgical complications and the application of MRI, compared to the VSB, the BB system is still pending FDA approval in the United States' market. Thus, the BB system may not be considered as a reimbursable item by a third party (e.g., healthcare insurance). Other disadvantages of the BB include possible postsurgical displacement of the BCI and extrusion of the implant, although this is uncommon. Lastly, the BB provides bilateral stimulation and thus may not be desirable when hearing in the other ear is normal; this is because there is a possible risk of signal interference to the normal contralateral ear. Careful assessment of individual preferences and preoperative trial on potential candidates with the Apollen bone conduction hearing aid provided by MED-EL could be helpful to determine its suitability before surgery is performed.

### 1.4. The Carina Fully Implantable Hearing Aid (Carina FIHA)

The Carina implantable hearing aid, developed by Otologics LLC (Boulder, CO, USA), was initially developed as a semi-implantable middle ear transducer (MET) but is now a fully implantable hearing device. The Carina device consists of the implant, the programming system, the charger, and the remote control. The implant itself contains the electronics which contain the microphone, battery, magnet, digital signal processor, and connector. The system utilizes a microphone located beneath the skin that picks up acoustic signals, which are then amplified and converted into an electrical signal. The signal is sent down the lead and into the transducer and the ossicular stimulator is coupled directly with the ossicular chain or the round window [8].

The Carina is considered to be another viable option for adults with moderate to severe hearing loss of conductive, sensorineural, or mixed etiologies. The device is CE-marked for sale and currently is still in the clinical trial stage, and therefore it has not been cleared for marketing by the FDA in United States. Three advantages of the Carina device include the following: (1) among the four devices discussed in this paper, the Carina is cosmetically the most appealing because no external processor can be seen; (2) it is easy to use; and (3) unilateral stimulation is provided. This device is thus suitable for adults with unilateral hearing loss such as unilateral congenital atresia. Siegert et al. investigated the surgical and audiological outcomes of Carina FIHA on five adults with either unilateral or bilateral congenital aural atresia [8]. All patients indicated no intra- or postoperative complications. Audiologically, all five patients demonstrated an average improvement of aided soundfield thresholds of approximately 35 dBHL with the Carina device. This provides the conclusion that the Carina device offers a new option for patients with congenital atresia [9].

However, there are several drawbacks of using the Carina device as follows: (1) it is not suitable for children and teenagers under 18 years of age; (2) the surgery is difficult to perform and it requires general anesthesia for about three hours; (3) revision surgery is required for battery change and future upgrade; (4) it is MRI-incompatible; (5) it is the most costly of all the MEIs; and (6) postoperative deterioration in hearing thresholds has been reported. Local experience in Hong Kong, with the application of the Carina device on five adults with sensorineural hearing loss, indicates a general drop of pure tone air conduction thresholds in all subjects postoperatively, at an average of 9 dB across the 250–8,000 Hz frequencies. A few cases also indicated a drop in bone conduction thresholds of about 10–15 dBHL [9]. Thus, preoperative counseling of the pros and cons of using a Carina device is suggested [9].

## 2. Candidacy

A candidate who has unilateral atresia must meet various criteria in order to be considered for implantation. In general, three types of considerations should be given: (1) the nature of the hearing loss, (2) patient preferences, and (3) issues related to practical usage.

**Figure 1:**
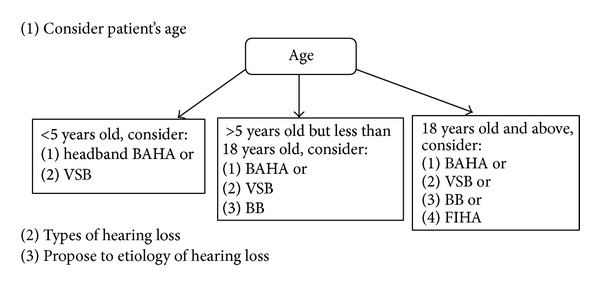
Flow chart on the clinical decision making process for implantable hearing devices.

In terms of the nature of the hearing loss, the patient should be selected according to the following criteria (see Figure 1 for the clinical decision making process).

- The air conduction hearing thresholds must fall within the manufacturer's suggested criteria, that is, not more than a moderate to severe degree of hearing loss, except for the FIHA, where bone conduction thresholds are used as one of the selection criteria. While the BAHA and the BB would allow hearing loss up to 45 to 55 dB HL, the VSB and the Carina would fit a hearing loss of up to 70 to 80 dB HL.
- The bone conduction threshold should be stable.
- Whether the middle ear structure would allow the coupling of the transducer.
- There should be an absence of retrocochlear pathology and auditory processing disorders.
- The unaided speech intelligibility should be better than 50% to 60%. However, there is no speech discrimination requirement for the BB device.

In regard to patient preferences, patient should understand the following factors (see Figure 1).

- The device is only for those who have do not receive satisfactory benefit from conventional bone conduction hearing aids or there is a cosmetic concern.
- There is limited evidence available for some of these devices.
- Only the BAHA and Carina are FDA- and/or CE-approved for conductive hearing loss and are eligible for reimbursement by third parties. Otherwise, the patient should be willing to pay for the device (i.e., VSB and BB).
- While local anesthesia is used for implanting the BAHA, general anesthesia surgical procedures are recommended for other devices and there are risks associated with the surgery.
- At the moment, the fact is only the BAHA and BB is MRI conditional, BB can have MRI up to 1.5 Tesla, while as long as the Baha sound processor is removed for the MRI procedure, a patient can have MRI up to 3 Tesla. The use of MRI is contraindicated with the VSB and Carina. The patient should understand that there could be issues with MRI assessment in the future.

In regard to the usage of implantable devices, the patient should be informed about the following.

- Due to normal hearing in the good ear, the benefits from amplification may be limited (e.g., amount of functional gain, speech intelligibility improvement).
- Due to bilateral stimulation by the implant, there could be a distortion of hearing.
- There could be implications related to long-term reliability of the device and other complications (e.g., skin overgrowth, extrusion of device, and device failure rate).
- Cosmetic appearance could be a concern with the BAHA.

## 3. Special Considerations in the Evaluation of an Atretic Ear

The consideration of an appropriate hearing device involves the examination of hearing thresholds. However, there are issues in regard to the measurement of air conduction thresholds in an ear with microtia and atresia because the calibration of the earphone is based on a normal physiologic pathway of air conduction. The volume of the ear under a supra-aural earphone is estimated to be about 6 cm^3^. However, this assumption cannot be made in cases of malformed external ear structures. Difficulties in establishing valid air conduction thresholds would also mean that we are not able to establish posttreatment advantages in air conduction thresholds. Thus, other types of outcome measures, such as soundfield thresholds measures, should be used.

When evaluating soundfield thresholds, the involvement of the good contralateral ear should be minimized, in order to obtain the true thresholds. When speech intelligibility is measured, the clinician should also consider whether the objective is to demonstrate the effects of implantation on hearing in the poor ear only (implant ear) or binaural hearing. In the former case, the good ear should be plugged and muffed to prevent its participation so that whether appropriate amplification has been provided to the poor ear can be verified. However, if the latter objective is desired, then the individual should be tested binaurally, without the good ear being plugged and muffed. Because of the good ear, it is likely that benefits in speech intelligibility will not be demonstrated if both speech and noise are being presented from the front loudspeaker. In other words, the effects of amplification may not be readily observable unless there is a spatial separation of speech and noise.

With the other ear having normal hearing, the benefits from an implant in the atretic ear are expected to be minimal. Thus, the above objective measures (e.g., soundfield air conduction testing and speech intelligibility evaluation) might not be effective in demonstrating benefits. Self-reports of aided benefit and satisfaction should be obtained to evaluate the outcomes.

## 4. Case Study

*Background.* K.C. is a 23-year-old man presenting with a unilateral microtia with congenital complete bony external auditory canal atresia on the right side, as confirmed by using a computer tomography (CT) scan. The malleus and incus had fused together and were found attached to the bony atretic plate. The stapes, oval window, and round window appeared normal on a CT scan. His major complaints were poor sound localization and speech understanding difficulties on the right side, especially in the presence of background noise. The unaided audiogram for K.C. was indicated in Figure 3.

**Figure 2:**
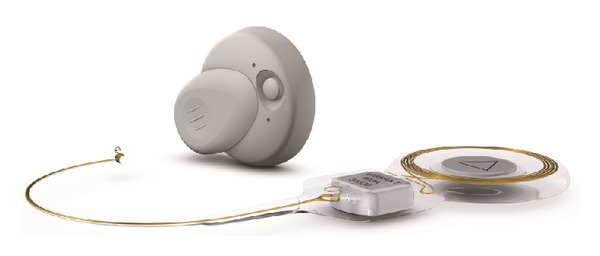
The Vibrant Soundbridge (VSB) implantable hearing device. The VSB system is comprised of two parts: the internal component is called the vibrating ossicular prosthesis (VORP) and the external component is an audio processor (AP). The VORP consists of a receiver coil, a magnet to hold the Amadé audio processor over the implant, a demodulator, the conductor link, and the floating mass transducer (FMT). The receiver coil sends the sound signal from the audio processor to the demodulator or electronics package. The demodulator demodulates the signal so that it can be converted into signals that will drive the FMT. The conductor link just sends these signals to the FMT. Adapted from marketing materials on the VSB, with permission of MED-EL, Innsbruck, Austria.

**Figure 3:**
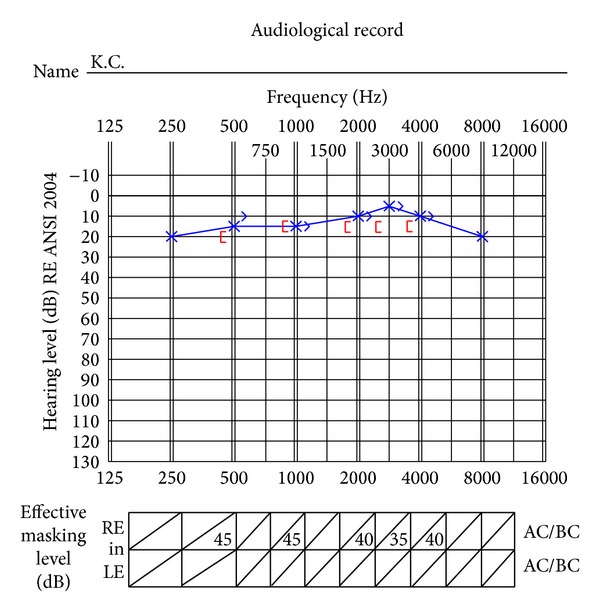
Unaided audiogram before surgery.

While both the VSB and Carina would be good choices, the VSB was chosen because K.C. was worried about possible future revision surgery in the case of a battery recharge problem. The VSB was an appropriate option for his right ear because CT scan findings showed that his stapes, oval window, and round window were normal and that therefore VSB could provide a unilateral stimulation to his right ear. Y.H. was counseled regarding the risks and benefits of the VSB (Figure 2). The surgery was performed in October 2012.

*Surgical Monitoring.* The operation was done under general anesthesia with facial nerve monitoring. The skin incision and soft tissue handling were such that the patient may need to undergo plastic reconstruction of the pinna at a later stage. Mastoidectomy was performed and access to the round window was achieved by removing atretic plate, malformed malleus and incus. The round window membrane needed to be fully exposed and this was performed by careful removal of bony overhang at the round window niche by a diamond burr. To ensure that the round window membrane will not be damaged by the FMT, a layer of perichondrium was placed on the round window membrane. To allow placement of the FMT in the round window niche, the titanium clip was removed. The FMT was carefully orientated and rested on the round window area. An extra perichondrium was put onto the FMT body to provide further stability.

Facial nerve monitoring is essential in this surgery for identifying any abnormal course of facial nerves and for avoiding facial nerve injury. Compound action potential (CAP) thresholds were examined during electrocochleography (ECochG) intraoperatively to determine the best site of the FMT placement as well as to evaluate the function of the implant system. With the electrodes placed on the promontory (active), vertex (reference), and forehead (ground), an increased CAP amplitude was observed with the FMT, and the goal was to achieve the best CAP thresholds possible. 

*Device Fitting.* The device was activated eight weeks after the surgery. This wait was required for wound healing both internally and externally. Based on the unaided soundfield hearing thresholds obtained from the implanted ear, the Amadé audio processor (MED-EL; Innsbruck, Austria) was programmed with the Connexx 6.11 software equipped with the Symfit database Rev.5.0. The default desired sensation level (DSL I/O) fitting formula was applied. Although the NAL/NL1 fitting formula is also available, it has not been modified to yield gain targets appropriate for a direct-drive device. When the aid was first switched on, the patient was not able to tolerate the prescribed gain. However, verification of the VSB device gain was difficult because the VSB is not an acoustic device. It is not possible to perform real ear measurements; soundfield aided thresholds, sentence reception thresholds (SRTs), and self-reports were obtained to evaluate the outcomes. K.C. returned for a fine-tuning session two weeks after device activation to ensure that he was listening at most comfortable levels as well as obtaining benefits from the device. Informal feedback on sound tolerance was obtained.

**Figure 4:**
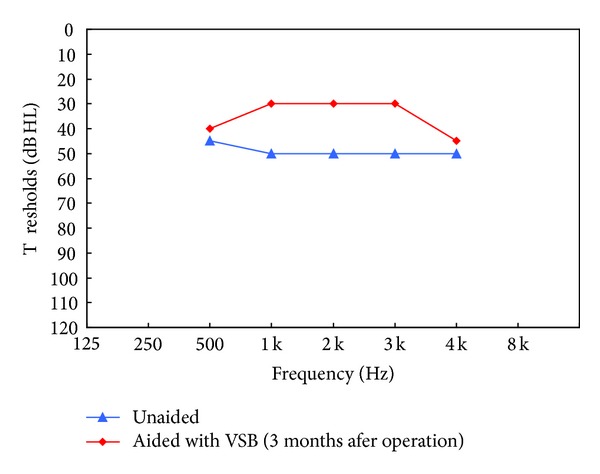
Unaided (U) and aided (A) soundfield audiogram obtained 3 months after the implantation of VSB in the right ear. Thresholds were measured with the warble tone signals presented from the poor ear (at 90 degree azimuths). The left (nonimplant) ear was plugged and muffed during testing.

*Results.* Change in residual hearing was evaluated by comparing the preoperative unaided bone conduction thresholds to the postoperative bone conduction thresholds. The results revealed no obvious change at any frequency.  Figure 4 presents unaided air and bone conduction thresholds before the surgery. The hearing thresholds were maintained after surgery. In addition, there were no postoperative complications observed or reported by K.C. after surgery. 

*Verification of the Implantable Hearing Device Performance.* One of K.C.'s chief preimplant complaints was his difficulty in picking up the signals from the right ear. In order to assess the aided benefits with the implant, aided soundfield thresholds were measured with warble tone signals presented at 90 degree azimuths, with the left ear both plugged and muffed. With the VSB set at the most comfortable listening level, it was also shown that VSB offered functional gains of 5 to 20 dB across the frequencies from 500 Hz to 4 kHz, when compared with the unaided thresholds (Figure 4). The amount of functional gain was less than that (45.5 dB) reported by Frenzel et al. [10]. However, the gain could not be adjusted to a higher volume setting because of intolerance. Aided thresholds ranged from 35 to 50 dBHL, which is slightly worse than those reported in the literature [10].

In the current case, we had aimed to present the results to demonstrate both the effects of amplification on the poor ear and binaural hearing. Speech reception thresholds (SRTs) were measured 3 months after implantation, when acclimatization, if any, would have been completed. With the good ear plugged and muffed, the SRT of the poor ear improved to 50.8 dB (A) in quiet, suggesting that although the VSB implant improved understanding of moderately soft speech, the benefit has not been optimized for soft speech. These results are consistent with findings in aided soundfield thresholds. SRT was −0.4 dB (A) in noise, thereby suggesting no improvement from the unaided condition. Thus, the implant improved the audibility of signals but not the S/N for 50% intelligibility when listening in noise.

**Figure 5:**
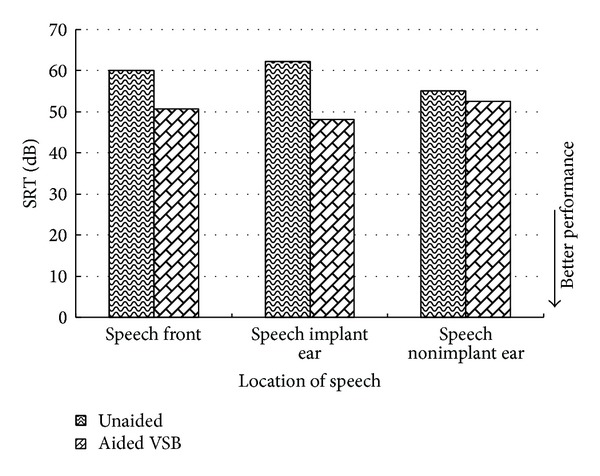
Speech reception thresholds (SRT) obtained in quiet using the Cantonese version of the Hearing In Noise Test (CHINT). SRTs were obtained unaided and aided at 3 months after activation of the VSB.

With binaural hearing in quiet, there was a slight elevation of SRT (0.8 to 3.8 dB) when compared to the unaided conditions (Figure 5). However, any changes smaller than 3.1 dB are within the confidence interval for test and retest [11], and therefore they could not be regarded as significant from a statistical point of view.

**Figure 6:**
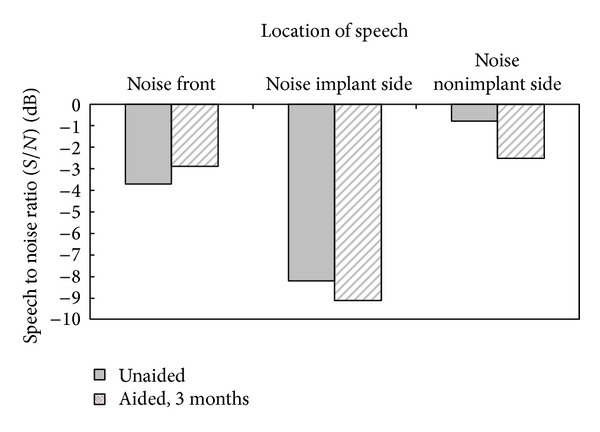
Speech reception thresholds (SRT) obtained in noise using the Cantonese version of the Hearing In Noise Test (CHINT). SRTs were obtained unaided and aided at 3 months after activation of the VSB.

In noise, as shown in Figure 6, the use of the VSB implant did not improve binaural squelch in the noise front condition; that is, the SRT did not improve compared to unaided, probably because the gain of the implant was low and thus did not yield true binaural hearing. When the noise was on the nonimplant side (good ear), the use of an implant-assisted speech reception (and thus SRT) improved to −2.5 dB S/N. Compared to when unaided, the patient was better able to take advantage of the spatial separation of speech and noise when noise was moved from the front to the implant side, resulting in an improvement of 6.2 dB in SRT. Overall, however, the changes in SRTs were small and could not be regarded as significant.

Self-reported aided benefit and satisfaction were measured with the two Chinese versions of the abbreviated profile of hearing aid benefit (APHAB) and the satisfaction with amplification in daily life (SADL) [12, 13]. The global score on the APHAB was 52 (with 100 indicating the greatest benefit) and on the SADL it was 4.29 (with 5 indicating the greatest satisfaction). Y.H. was satisfied with the VSB middle ear implant in his right ear, although the benefits were reportedly only moderate. However, these findings are not unexpected, given that the contralateral ear exhibits normal hearing ability. The patient also reported good sound quality and comfort and he accepted the appearance of the aids as was often reported in previous studies [10]. Y.H. used the VSB for approximately 4 to 5 hours per day, primarily for his part-time work and for communication at home. His ability to hear the signals from the right ear (the poor ear) reportedly improved his localization ability, despite the distorted interaural phase and time differences due to the use of a hearing device in one ear.

## 5. Discussion

Overall, there are many practical concerns when making clinical decisions on the selection of an appropriate implantable device for an adult with unilateral atresia. One of the most important questions to ask is the age of the patient (see Figure 1). The second question will be the types of hearing loss and thirdly it would be the etiologies of hearing loss.

For microtia patient with pure conductive hearing loss, the underlying cause is likely due to malformed ossicles; then the option of VSB surgery should only be considered after radiological studies where an appropriate coupling is possible. The options of BAHA and BB do not have this constraint. However, the clinician should be aware that both the BAHA and BB provide bilateral stimulations and thus they may introduce interference to the contralateral ear. In contrast, the VSB and Carina FIHA provide a unilateral stimulation of the worse ear. Skin tract problem of BAHA must be thoroughly discussed. Also the VSB and FIHA are neither MRI-incompatible nor FDA-approved, and the potential candidates should be well informed of the risks including worsening of hearing loss and implant failure before they opt for a relatively difficult operating procedure in VSB and FIHA.

Regarding the cost, the FIHA is the most expensive in comparison with the VSB and BB. Thus, preoperative counseling of the risks and benefits of each device is important.

## 6. Conclusion

Despite the lack of high-level evidence about these devices, the selection of appropriate implantable hearing devices should be based on various factors, including radiological findings and patient preferences. In addition, surgical complications, whether the device is FDA-/CE-approved, and the finances should be considered. To ensure the accurate evaluation of candidacy and outcomes, the evaluation methods should be adapted to suit the type of hearing device.
